# A flexible network-based imputing-and-fusing approach towards the identification of cell types from single-cell RNA-seq data

**DOI:** 10.1186/s12859-020-03547-w

**Published:** 2020-06-11

**Authors:** Yang Qi, Yang Guo, Huixin Jiao, Xuequn Shang

**Affiliations:** School of Computer Science, Northwestern Polytechnical University, Xi’an, 710072 China

**Keywords:** scRNA-seq, Dropout events, Biological networks, Data integration, Cell types

## Abstract

**Background:**

Single-cell RNA sequencing (scRNA-seq) provides an effective tool to investigate the transcriptomic characteristics at the single-cell resolution. Due to the low amounts of transcripts in single cells and the technical biases in experiments, the raw scRNA-seq data usually includes large noise and makes the downstream analyses complicated. Although many methods have been proposed to impute the noisy scRNA-seq data in recent years, few of them take into account the prior associations across genes in imputation and integrate multiple types of imputation data to identify cell types.

**Results:**

We present a new framework, NetImpute, towards the identification of cell types from scRNA-seq data by integrating multiple types of biological networks. We employ a statistic method to detect the noise data items in scRNA-seq data and develop a new imputation model to estimate the real values of data noise by integrating the PPI network and gene pathways. Meanwhile, based on the data imputed by multiple types of biological networks, we propose an integrated approach to identify cell types from scRNA-seq data. Comprehensive experiments demonstrate that the proposed network-based imputation model can estimate the real values of noise data items accurately and integrating the imputation data based on multiple types of biological networks can improve the identification of cell types from scRNA-seq data.

**Conclusions:**

Incorporating the prior gene associations in biological networks can potentially help to improve the imputation of noisy scRNA-seq data and integrating multiple types of network-based imputation data can enhance the identification of cell types. The proposed NetImpute provides an open framework for incorporating multiple types of biological network data to identify cell types from scRNA-seq data.

## Background

The advance of single-cell RNA sequencing (scRNA-seq) technologies nowadays provides good opportunities to comprehensively investigate the transcriptome-wide variability and cell heterogeneity at the single-cell resolution [1–3]. Unlike the bulk-cell RNA sequencing, which performs high-throughput sequencing of RNA refined from millions of cells and the expression of each gene would be averaged across cells [4, 5], scRNA-seq performs sequencing of RNA refined from a single cell and the expression of genes reflect the transcriptomic characteristics at the single-cell level. However, scRNA-seq data usually have relatively higher noise than the bulk-cell RNA sequencing data due to the low amounts of transcripts in single cells and sequencing technical biases [6, 7]. The most well-known noise type in scRNA-seq data is the dropout events, where a gene actually expressed even at a high level but was not detected in sequencing due to the limitation of technical sensitivity [8, 9]. The dropout events can be deemed as a special type of false zeros in data. In addition, data noise may stochastically occur at systematical level, even for the gene with high expression level, due to the technical biases [10, 11]. Therefore, it is crucial to develop computational methods to address the noise issues at both low-expression and high-expression levels in scRNA-seq data in order to facilitate the downstream researches on scRNA-seq data, such as the identification of cell types [12, 13], differential gene expression analysis [14, 15] and characterization of dynamic profiles in rare cell types [16], etc.

Many computational methods for analysing scRNA-seq data have been developed in recent years from different perspectives, such as imputing the dropout events in scRNA-seq data [4, 17, 18], identifying cell types from scRNA-seq data [12, 19] and detecting rare cell types [20], etc. To address the dropout events in scRNA-seq data, SAVER [17] incorporates the similarity information across genes to impute the real expression of genes by using a Bayesian approach. MAGIC [18] imputes the gene expression of missing values based on similar cells using a network diffusion approach. However, both SAVER and MAGIC estimate the expression values of all genes, and this may alter the expression values which are not affected by the dropout events [4], so they would potentially introduce new biases into the data. Besides, scImpute [4] only imputes the missing values with high dropout probability based on similar cells and does not alter the values which are deemed as real expression items. DrImpute [21] estimates the real values of dropouts by averaging the corresponding gene expression from different clustering results. RESCUE [22] considers the challenge of dropout effect on the cell-clustering using all genes and uses a bootstrap strategy to select gene features to promote the robustness of cell-clustering to improve the data imputation. Although those imputation methods have been demonstrated to be effective in handling the dropout events to some extent, there are at least two drawbacks that need to be further addressed at present. Firstly, most existing methods only consider the cell similarity in dropout imputation, but ignore the associations across genes. However, there usually exist associations between genes in terms of their biological functions or regulation mechanisms. It is necessary to consider these associations between genes in dropout imputation. Secondly, most existing methods mainly focus on the imputation of dropout noise at low-expression level, while the stochastic noise may also occur at high-expression level due to the technical biases [10, 11]. It is essential to impute the data noise not only at low-expression level but also at high-expression level in handling the noisy scRNA-seq data.

In addition, one of the most important analysis missions on scRNA-seq data is to identify cell types by taking the noise effect into account. CIDR [19] is the first clustering method to identify cell types by considering the dropout events. While it cannot be used as an imputation method in general since the imputed values are not stable when one cell is paired up with different cells [4, 19]. In general, we can use the dropout imputation methods to perform de-noise on raw scRNA-seq data, and then identify cell types based on the imputed data. While it is hard to consider multiple types of prior biological knowledge in the identification of cell types based on scRNA-seq data. Actually, other types of biological data can be used to guide the imputation of scRNA-seq data, such as gene functional networks and gene pathways, etc., and thus help to enhance the identification of cell types. It is still necessary to develop more flexible and accurate methods to impute the noisy scRNA-seq data, thus to improve the accuracy of downstream analyses.

In this paper, we propose a new framework, so-called NetImpute, towards the identification of cell types from scRNA-seq data by integrating multiple types of biological networks. We first impute the noisy raw scRNA-seq data by incorporating multiple types of biological networks to obtain different types of imputation data. Then, we integrate multiple imputation data to identify cell types according to the hierarchical clustering algorithm. Specifically, the overall NetImpute framework includes two main models: the imputation model and the integration model. In the NetImpute imputation model, we take into account the gene associations from the PPI (Protein-Protein Interaction) network and gene pathways to impute the noise data items in scRNA-seq data by training a series of regression models based on cell similarity information, thus to obtain different types of imputation data. Meanwhile, we utilize a statistic method based on Chebyshev inequality [23, 24] to detect the noise data items in scRNA-seq data. In the NetImpute integration model, we fuse the similarity information across cells from both the PPI-based and pathway-based imputation data to identify cell types using the hierarchical clustering algorithm. Comprehensive experiments based on three real data demonstrate that: (1) The proposed network-based imputation model can estimate more accurate values of noise data items, and thus help to improve the cell typing from scRNA-seq data. (2) Integrating multiple types of imputation data can help to further improve the performance of identifying cell types from scRNA-seq data.

The main contributions of this study can be summarized as follows: (1) We propose a new imputation model to estimate the real values of noise data items in scRNA-seq data by taking into account the association information across genes based on biological networks. (2) We propose a new statistic method based on Chebyshev inequality to detect noise data items at both low-expression and high-expression levels and consider the both types of noise in imputation. (3) We propose a new method integrating multiple types of imputed data using different biological networks to identify cell types from scRNA-seq data.

## Methods

In this section, we introduce the proposed network-based imputation method and multiple imputation data fusion framework towards the identification of cell types from scRNA-seq data.

### Problem overview and computational framework

**Fig. 1:**
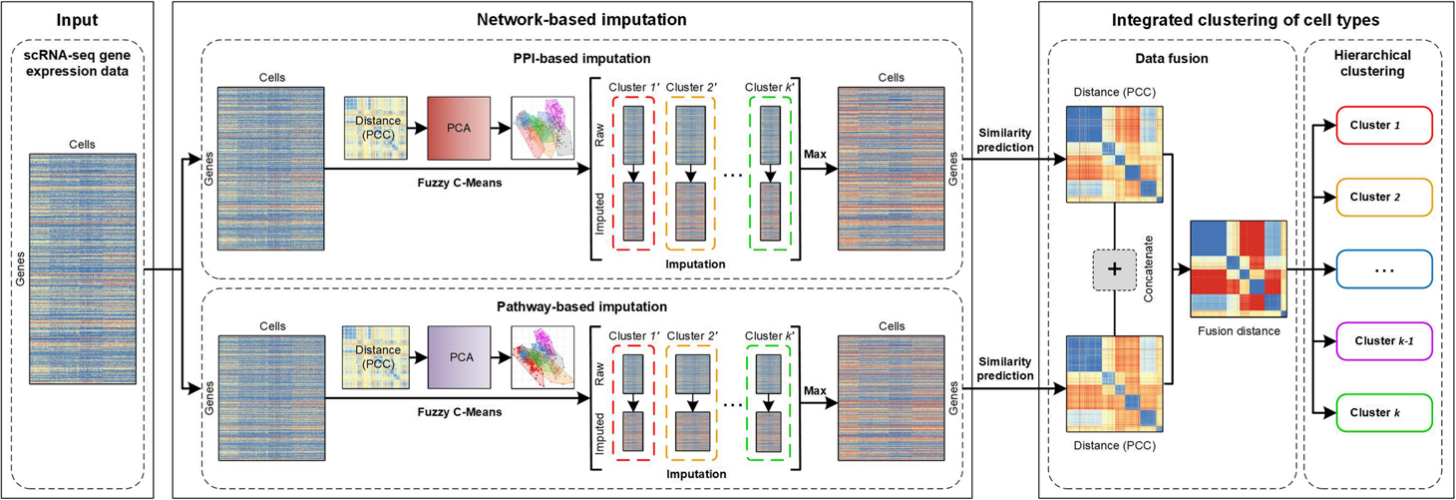
The overall workflow of NetImpute framework. The network-based imputation model integrates biological networks (PPI network and gene pathways) to impute noise data items in raw scRNA-seq data. The integrated clustering model fuses multiple types of imputation data to identify cell types according to the hierarchical clustering algorithm

As presented above, there are two main issues may affect the identification of cell types from scRNA-seq data at present. Firstly, scRNA-seq data include higher noise than the bulk RNA-seq data due to the dropout events and high variability in technical replicates [4, 6, 7]. The dropout events produce plenty of false-positive zero values in scRNA-seq data since the low RNA input in sequencing and the stochastic noise of gene expression in individual cells. Besides the dropout events, technical noise in scRNA-seq also affects the variability of gene expression, even for the high level of expression data [10, 11]. These noise items affect the accuracy of cell-types identification on scRNA-seq data. Secondly, only gene expression information may not enough to identify cell types accurately from noisy scRNA-seq data, and the incorporation of multiple types of prior biological information hopes to improve the accuracy of cell-types identification. In this paper, we present a new computational framework, which is so-called NetImpute, to address the issues mentioned above by imputing noise values via incorporating multi-type biological networks. In particular, we first incorporate the PPI network and gene pathways to impute noise values from dropout events and high expression biases in scRNA-seq data respectively, and then we integrate the imputation data based on multi-type biological networks to identify cell types from scRNA-seq data. In summary, the main steps of NetImpute include: (1) PPI network-based imputation for scRNA-seq data. (2) Pathway-based imputation for scRNA-seq data. (3) Imputation data integration and cell-types identification. Figure 1 shows the illustration diagram of the overall framework of NetImpute. We introduce the details of each step respectively in the following sections.

### Network-based imputation

#### Detection of fuzzy cell subpopulations and outliers

We impute the dropout and bias values in scRNA-seq data based on the reliable expression information of similar cells in subpopulations and gene association knowledge in biological networks. It is crucial to identify similar cell subpopulations before data imputation. However, as the existence of dropout events and data biases in high-level expression, it is difficult to directly estimate accurate cell subpopulations on scRNA-seq data. Considering the uncertainty of clustering cell subpopulations based on the raw scRNA-seq data, we preliminarily use the fuzzy clustering method to identify fuzzy cell subpopulations, in which a cell can belong to more than one cluster in general. Specifically, let *X*_*m*×*n*_ be the raw scRNA-seq data, where *m* is the number of genes (rows) and *n* is the number of cells (columns). We first calculate the Pearson distance matrix *D*_*n*×*n*_ between cells (PCC based distance), then the principal component analysis (PCA) is performed on *D*_*n*×*n*_ and the reduction output matrix is denoted as *Z*_*p*×*n*_. The number of conserved principal components *p* is decided by calculating the decay rate of the explained variance between two consecutive components. We require the variance decay rate between two consecutive components no less than 0.6 and 3≤*p*≤10 in practice. Based on *Z*_*p*×*n*_, we utilize the fuzzy c-means (FCM) algorithm [25, 26] to cluster all cells into *k* subpopulations, in which a cell sample can belong to multiple subpopulations. In particular, the FCM algorithm can predict the probability of each cell belongs to the *i*-th cluster (cell subpopulation). For each cell sample *S*_*j*_, we assign *S*_*j*_ to the unique cluster *C*_*i*_ if the possibility of *S*_*j*_∈*C*_*i*_ is greater than 0.5, otherwise we assign *S*_*j*_ to those clusters if the possibility of *S*_*j*_∈*C*_*i*_ is range from 2/*k* to 0.5. We assume the samples which have not assigned to any clusters as outliers and remove them from the sample list in the downstream data imputation.

#### Identification of noise data items at the low-expression and high-expression levels

Once we obtain the preliminary subpopulations of cells, the next step is to identify intra-cluster gene expression noise in each subpopulation. As previous studies in [4, 21], we assume that the genes in the same cell subpopulation have roughly similar expression patterns. The gene expression which seriously deviates from the average expression of the gene in a cell subpopulation is deemed to have high possibility to be a noise item and needs to be imputed. Since the noise data items include the deviated gene expression at both low-expression and high-expression levels, the dropout events are automatically attributed to the low-expression noise in our research. Meanwhile, we also consider the high-expression noise data in imputation. To identify the noise data items of gene expression in a subpopulation, we utilize the Chebyshev inequality [23, 24] based statistic method to distinguish the noise data from the background expression of genes in a subpopulation.

Let the expression of gene *i* in cell subpopulation *k* to be a variable $X_{i}^{(k)}$, the expectation of $X_{i}^{(k)}$ is $E (X_{i}^{(k)})= \hat {\mu }_{i}^{(k)}$ and the variance is $D (X_{i}^{(k)})= {\hat {\sigma }_{i}^{(k)}}{^{2}}$, for any *ε*>0, according to the Chebyshev inequality theorem, there is,

1 $$ P \{ | X_{i}^{(k)}-\hat{\mu }_{i}^{(k)} | < \varepsilon \}\geq 1-\frac{{\hat{\sigma}_{i}^{(k)}}{^{2}}}{\varepsilon^{2}}  $$

Equation 1 gives the lower bound of $P \{ | X_{i}^{(k)}-\hat {\mu }_{i}^{(k)} | < \varepsilon \}$ for any *ε*>0. Since there is no limitation to the distribution of variable $X_{i}^{(k)}$ in the Chebyshev inequality theorem, it is applicable for any variables of genes in each cell subpopulation. Specifically, when $\varepsilon = \sqrt {2}\hat {\sigma }_{i}^{(k)}, 1-{\hat {\sigma }_{i}^{(k)}}{^{2}}/\varepsilon ^{2}= 0.5$, this defines the lower bound of $P \{ | X_{i}^{(k)}-\hat {\mu }_{i}^{(k)} | < \varepsilon \}$ is 0.5. ${\hat {\sigma }_{i}^{(k)}}{^{2}}$ is the expression background variance of gene *i* in subpopulation group *k*. Similar to [24], we define the expression variance of gene *i* on cell *j* in subpopulation *k* as ${\sigma _{i,j}^{(k)}}{^{2}}$, where ${\sigma _{i,j}^{(k)}}{^{2}}= (X_{i,j}^{(k)}-\hat {\mu }_{i}^{(k)})^{2}$. If ${\sigma _{i,j}^{(k)}}{^{2}} \leq {\hat {\sigma }_{i}^{(k)}}{^{2}}$, the expression variance of $X_{i,j}^{(k)}$ is not greater than the background variance of gene *i* in subpopulation *k*, $X_{i,j}^{(k)}$ is more likely to be a credible expression data and does not need to be imputed. Otherwise, if ${\sigma _{i,j}^{(k)}}{^{2}}> {\hat {\sigma }_{i}^{(k)}}{^{2}}$, the expression value $X_{i,j}^{(k)}$ has high possibility to be a noise data item and it will be selected as a candidate item that needs to be further imputed. However, it is inflexible to define the threshold as a certain value ${\hat {\sigma }_{i}^{(k)}}{^{2}}$ at both the low-expression and high-expression levels. In fact, in most data analyses, we hope to flexibly define the selection thresholds of noise data items at the low-expression and high-expression levels respectively, and thus to control the fraction of imputation to satisfy different analysis missions. In addition, it is necessary to define different expression variances for the low-expression and high-expression noise according to adaptive thresholds in various data distributions. To overcome the inflexible issue in threshold selection, we adopt an adaptive method, which was first proposed in image processing [24], to define the discrimination thresholds based on the background variance in a specific subpopulation. Based on Eq.1, when fixing the $\varepsilon, {\sigma _{i,j}^{(k)}}{^{2}}\leq {\hat {\sigma }_{i}^{(k)}}{^{2}}$ can be estimated by $1-{\sigma _{i,j}^{(k)}}{^{2}}/\varepsilon ^{2}\geq 1-{\hat {\sigma }_{i}^{(k)}}{^{2}}/\varepsilon ^{2}$; rather, ${\sigma _{i,j}^{(k)}}{^{2}}> {\hat {\sigma }_{i}^{(k)}}{^{2}}$ can be estimated by $1-{\sigma _{i,j}^{(k)}}{^{2}}/\varepsilon ^{2}< 1-{\hat {\sigma }_{i}^{(k)}}{^{2}}/\varepsilon ^{2}$. In our situation, when giving a fixed *ε*, we want to detect the noise data items which have variance ${\sigma _{i,j}^{(k)}}{^{2}}> {\hat {\sigma }_{i}^{(k)}}{^{2}}$, so the problem is equivalent to $1-{\sigma _{i,j}^{(k)}}{^{2}}/\varepsilon ^{2}< 1-{\hat {\sigma }_{i}^{(k)}}{^{2}}/\varepsilon ^{2}$. For each data $X_{i,j}^{(k)}$ in subpopulation *k*, we calculate the value of discrimination function $D (X_{i,j}^{(k)})= 1-{\sigma _{i,j}^{(k)}}{^{2}}/\varepsilon ^{2}< T$ by fixing $\varepsilon = \sqrt {2}\hat {\sigma }_{i}^{(k)}$, to decide whether it is a noise data item. In theory, $T= 1-{\hat {\sigma }_{i}^{(k)}}{^{2}}/ \varepsilon ^{2}$ is the strict upper bound threshold of $D (X_{i,j}^{(k)})$ in discrimination. We also define an adaptive threshold of $D (X_{i,j}^{(k)})$ by relaxing the upper bound as $T= 1-{\hat {\sigma }_{i}^{(k)}}{^{2}}/\varepsilon _{t}^{2}$, where $\varepsilon _{t}= \varepsilon \pm \theta \hat {\sigma }_{i}^{(k)}, 0< \theta < 1$. Since *ε*_*t*_ is tuned according to ${\hat {\sigma }_{i}^{(k)}}$, the threshold of *T* can be adapted by the data background variance once giving a predefined parameter *θ*. In addition, according to Eq.1, we can test the noise data items at both the low-expression and high-expression levels. In order to consider the situation that the dropout events are the main noise data items in the low-expression aspect in scRNA-seq data, we define different values of *T* to detect the noise data items in the low-expression and high-expression aspects respectively as [24],

2 $$ T = \left\{\begin{array}{cc} \frac{0.5+ (1 -\frac{{\hat{\sigma }_{i}^{(k)}}{^{2}}}{\varepsilon_{1}^{2}})}{2}, & X_{i,j}^{(k)}\leq \hat{\mu }_{i}^{(k)} \\ \frac{0.5+ (1 -\frac{{\hat{\sigma }_{i}^{(k)}}{^{2}}}{\varepsilon_{2}^{2}})}{2}, & X_{i,j}^{(k)}> \hat{\mu }_{i}^{(k)} \end{array}\right.  $$

where $\varepsilon _{1}= \varepsilon -\theta _{1}\hat {\sigma }_{i}^{(k)}$ and $\varepsilon _{2}= \varepsilon -\theta _{2}\hat {\sigma }_{i}^{(k)}, \varepsilon = \sqrt {2}\hat {\sigma }_{i}^{(k)}, \hat {\mu }_{i}^{(k)}= \sum _{j=1}^{N}X_{i,j}^{(k)}/N, 0< \theta _{1},\theta _{2}< 1$. In this study, we set *θ*_1_=0,*θ*_2_=0.5 as the default values. If $X_{i,j}^{(k)}\leq \hat {\mu }_{i}^{(k)}$, the data point $X_{i,j}^{(k)}$ belongs to the low-expression aspect of gene *i* in subpopulation *k*, otherwise $X_{i,j}^{(k)}$ belongs to the high-expression aspect of gene *i* in subpopulation *k*.

Specifically, for each gene expression data item *X*_*i*,*j*_ in subpopulation *k*, denoted as $X_{i,j}^{(k)}, {\sigma _{i,j}^{(k)}}{^{2}}= (X_{i,j}^{(k)}-\hat {\mu }_{i}^{(k)})^{2}$ is the variance of $X_{i,j}^{(k)}$ in subpopulation *k*, we can judge whether $X_{i,j}^{(k)}$ is a noise item by,

3 $$ D (X_{i,j}^{(k)})= 1-\frac{{\sigma_{i,j}^{(k)}}{^{2}}}{\varepsilon^{2}}< T  $$

where $\varepsilon = \sqrt {2}\hat {\sigma }_{i}^{(k)}$. Meanwhile, if $X_{i,j}^{(k)}\leq \hat {\mu }_{i}^{(k)}, X_{i,j}^{(k)}$ is a low-expression noise item, otherwise $X_{i,j}^{(k)}$ is a high-expression noise item in subpopulation *k*.

#### Imputation of noise data based on biological networks

After identifying the noise items in each preliminary cell subpopulation in scRNA-seq data, we impute these items with the aid of biological networks. We suppose that the expression of a gene is affected by their neighborhood genes in the biological network. Therefore, for the noise items of a gene in one specific fuzzy cell subpopulation, we first learn a regression model based on the network neighborhood genes’ expression data which have high confidence to be correct values in the corresponding cell subpopulation. Then we use the learned regression model to estimate the real values of the gene’s noise items by integrating the expression of its neighborhood genes in biological network. In particular, we first identify all noise data items in each fuzzy cell subpopulation and borrow the information of genes which have accurate values with high confidence to predict the real values of noise data items. Let *X*_*i*,*j*_ be the expression of gene *i* on cell *j* in the fuzzy subpopulation *k*, *N*_*i*_ is the neighborhood gene set of gene *i* in the biological network *G* and *A*_*i*_ is the set of cells in the subpopulation *k* which have high confidence values for gene *i*. To learn the expression associations between gene *i* and its neighborhood genes *N*_*i*_ in *G*, we use the regularized non-negative least squares (NNLS) regression model [27, 28] as,

4 $$ \begin{aligned} \hat{\beta }^{(i)}= &\underset{\beta^{(i)}}{\text{argmin}} \{ \| X_{i, A_{i}}- \beta^{(i)} X_{N_{i}, A_{i}} \|_{2}^{2} +\lambda \alpha \| \beta^{(i)} \|_{1}\\ &+\frac{1}{2}\lambda (1-\alpha) \| \beta^{(i)} \|_{2}^{2} \}\\ &\!\!\!\!\!\!\!\!\!\!\!\!\!\!\!\!\!\!\!\!\!\!\!\!\!\!\!\! \text{subject to},\beta^{(i)}\geq 0,\lambda \geq 0,0\leq \alpha \leq 1 \end{aligned}  $$

Recall that $X_{i, A_{i}}\in \mathbb {R}^{| A_{i} |}$ represents the vector of expression values of gene *i* in all cells of *A*_*i*_, which have high confidence expression values in the subpopulation *k*. $X_{N_{i},A_{i}}\in \mathbb {R}^{| N_{i} |\times | A_{i} |}$ is a sub-matrix in the raw expression data, where the row and column coordinates are respectively in *N*_*i*_ and *A*_*i*_. *β*^(*i*)^ is the coefficient vector with length |*N*_*i*_|. *λ* is the coefficient for both L1 and L2 regularization items, *α* is a parameter to weight the effect of L1 and L2 regularization in order to obtain a sparse estimated $\hat {\beta }^{(i)}$. Considering there may be many neighborhood genes for most genes in the biological network, the imputation regression model is affected by many neighborhood genes. We want to obtain a sparse model for most genes to ease the overfitting in learning. In this study, we set *λ*=10 and *α*=0.5 in default. Finally, the learned imputation model is used to impute the real expression of gene *i* on cell *j*,

5 $$ \hat{X}_{i,j} = \left\{\begin{array}{cc} X_{i,j}, & j\in A_{i} \\ \hat{\beta }^{(i)}X_{N_{i},j}, & j\notin A_{i} \end{array}\right.  $$

**Fig. 2:**
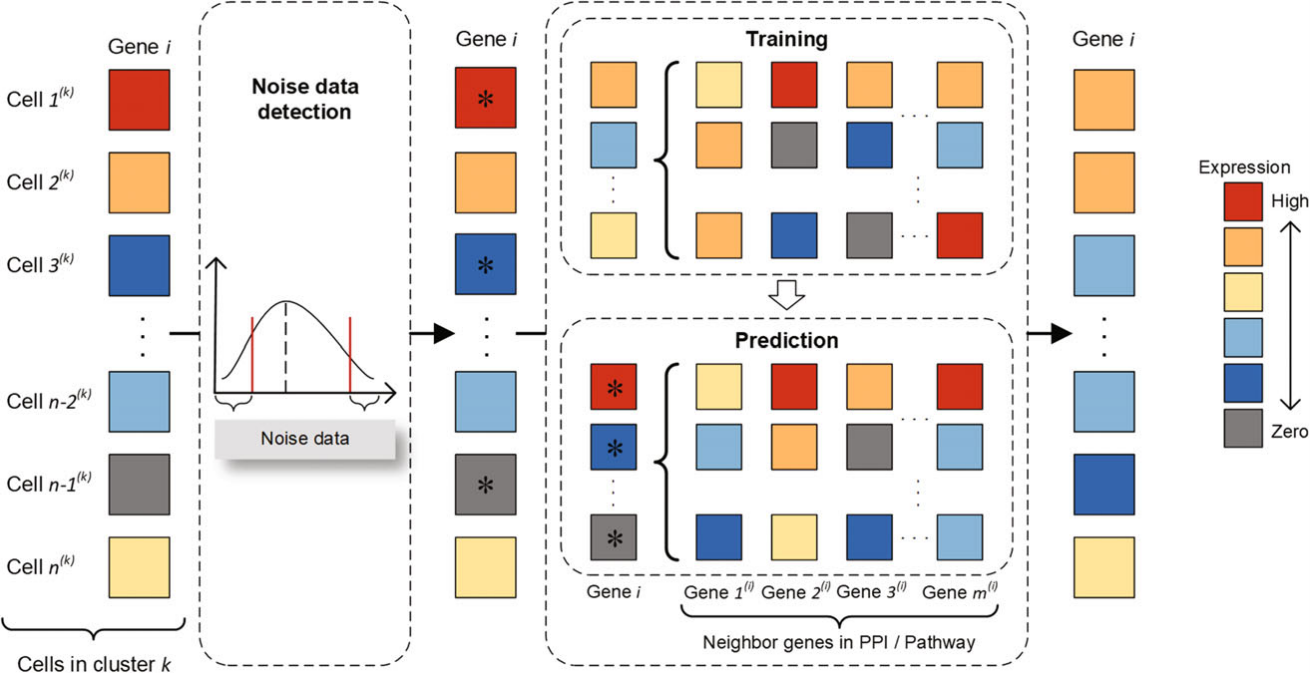
Overall procedure of the network-based imputation model in NetImpute framework. The statistic method is used to identify noise data items at both the low-expression and high-expression levels. For each gene’s noise items (star), NetImpute integrates the neighborhood genes in networks (PPI network or gene pathways) that have high-confidence expression values in a specific fuzzy cell subpopulation to train a regression model, and then the learned regression model is used to impute the noise data

In each cell subpopulation *k*, we construct a separate regression model for each gene *i* by incorporating the associations across genes in the biological network to impute the correct values of noise data items. Figure 2 shows the overall procedure of the proposed imputation model that estimates the correct values of noise data items for a gene. Based on each initial fuzzy subpopulation of cells, one noise data item for a gene will have an estimated value. According to all fuzzy subpopulations of cells in the raw scRNA-seq data, one noise data item for a gene may have more than one imputed value since there are overlaps in the fuzzy subpopulations of cells. Therefore, for each noise data item which may have multiple imputation values, we assign its final value as the maximum one. In addition, different numbers of initial fuzzy cell subpopulations may affect the final estimation of noise data items, since the imputation independently performs on each fuzzy cell subpopulation. After testing on various types of real data, we found that setting the number of fuzzy cell subpopulations close to the true number of cell-type clusters hopes to obtain more accurate imputation data for the noise data items. Therefore, we recommend setting the number of initial fuzzy subpopulations of cells roughly close to the true number of cell-type clusters in practice before using the NetImpute for imputation.

### PPI network-based imputation

We introduce the PPI network to obtain the association information between genes, and thus to impute the raw scRNA-seq data using the proposed imputation model. To ensure each gene has neighborhood genes for reference in imputation, we select the genes which interact with at least two genes in PPI network. In addition, to acquire more confident gene expression data, we filter out the genes which have zero values in more than 90% cells from the raw scRNA-seq data and obtain the expression data of the overlap genes between the conserved genes in raw scRNA-seq data and the PPI network to perform the downstream imputation. We set the initial fuzzy subpopulation clusters of cells in raw scRNA-seq data close to the true number of cell clusters in the PPI network-based imputation.

### Pathway-based imputation

We also incorporate the gene pathway information to consider the biological regulated associations between genes to impute the raw scRNA-seq data by using the proposed imputation model. A gene pathway usually describes a separate gene regulation unit in biological metabolism and it appears as a directed subgraph in the data structure. To consider more complete gene regulation information, we ignore the specific directed interactions in each pathway by treating a pathway as a complete interaction subgraph between the corresponding genes. We collect all available pathways together to obtain all candidate pathway genes. In addition, to acquire more confident gene expression data, we also filter out the genes which have zero values in more than 90% cells from the raw scRNA-seq data. Finally, we obtain the expression data of overlap genes between the conserved genes in raw scRNA-seq data and the candidate pathway genes to perform the downstream imputation. Since a gene may attend multiple pathways in biological regulation, so one noise data item of a gene may obtain multiple imputation values according to different pathways. To obtain the unique imputation, we select the maximum value as its final imputation data if a noise data item has multiple imputation values. Specifically, suppose gene *i* attends *L* pathways, $N_{i}^{l}$ is neighborhood gene set of gene *i* in the *l*-th pathway and $\hat {\beta }_{l}^{(i)}$ is the coefficients of the corresponding regression model based on the *l*-th pathway, the final imputation value of the noise data item $\hat {X}_{i,j}$ of gene *i* on cell *j* is,

6 $$ \hat{X}_{i,j}= \underset{l}{\text{max}} \{ \hat{\beta }_{l}^{(i)}X_{N_{i}^{l},j} \}  $$

### Imputed data fusion and cell-types prediction

We integrate the PPI network and gene pathway information to impute the raw scRNA-seq data respectively and then fuse the imputed data to identify cell types from scRNA-seq data. Based on the PPI network and gene pathways imputed data, we first calculate the Pearson distance matrixes between cells and denote them as *M*_1_ and *M*_2_ respectively. To combine the similarity information of cell samples from both the PPI network-based and the pathway-based imputation data, we simply concatenate the distance matrixes *M*_1_ and *M*_2_ as the combined distance feature matrix of cell samples. Then, we calculate the integrated similarities of cell samples based on the combined feature matrix. We assume that a pair cells are similar if they have similarly pairwise neighborhood features. In detail, we respectively calculate the Pearson distance matrix *Dist*(*P*) and Spearman distance matrix *Dist*(*S*) between cell samples, and then we obtain the integrated similarity distance matrix between cells by,

7 $$ Dist (M)= \frac{Dist (P)+Dist (S)}{2}  $$

Finally, based on the integrated similarity distance matrix between samples, we use the hierarchical clustering algorithm [29] to predict cell subpopulations by cutting the dendrogram based on different distance heights to obtain more accurate cell-type clusters. Figure 1 shows the overall procedure of integrated cell-types clustering.

## Results

### Datasets and data processing

To evaluate the effectiveness of the proposed imputation method-NetImpute, we used three public scRNA-seq datasets in the Gene Expression Omnibus (GEO) database in our experiemnts (also collected by https://hemberg-lab.github.io/scRNA.seq.datasets). These three scRNA-seq datasets include: (1) The scRNA-seq data on differentiation of human cerebral organoid cells (GSE75140), which was so-called Camp data [30], and 5 initial cell types were annotated on cells; (2) The scRNA-seq data on the cells of human brain (GSE67835), which was so-called Darmanis data [31], and 8 initial cell types were annotated on cells; (3) The scRNA-seq data on the cells of human colorectal tumors (GSE81861), which was so-called Li data [32], and 9 initial cell types were annotated on cells. The human PPI network data was downloaded from the PICKLE website [33, 34], where the data was collected from multiple public databases [35–40], which include 15,434 proteins and 161,007 interactions between proteins (PICKLE 2.2). We used the Retrieve/ID mapping tool from UniProt [41, 42] mapped the gene identities to symbols, and obtained 15,336 genes and 160,857 interactions between genes. The gene pathway data was downloaded from the Broad Institution database [43, 44], which includes 5,266 genes in 186 different gene pathways.

In order to be convenient for the evaluation of methods, we removed the cell samples which have unknown cell-type labels. Meanwhile, the genes that have zero values on more than 90% cells were also filtered out. Finally, in the PPI network-based and gene pathway-based imputation analyses, we used the expression data of overlap genes between the processed scRNA-seq data and the PPI network/pathways genes. Table 1 gives the basic statistics information of the data used in our experiments.

**Table 1:** The basic statistics of the datasets

Dataset	Camp	Darmanis	Li
Cells	553	420	561
Genes (PPI-based)	7,856	8,335	11,049
Genes (Pathway-based)	2,520	2,879	3,715

### NetImpute recovers the low-expression and high-expression noise data items

The proposed NetImpute method aims to impute the noise data items in scRNA-seq data by borrowing gene association information from biological networks. As there are not ground truth in scRNA-seq data can be used to validate the confidence of the imputed data. In order to test the performance of NetImpute on the estimate of noise data items, we first investigate the imputation performance based on the simulation data. Since the NetImpute method can handle the noise data at both low-expression and high-expression levels, we simulated different types of noise data and used NetImpute to recover the real values of them by incorporating gene association information from the PPI network and gene pathways respectively. Specifically, to generate different types of simulation data, we selected the genes which have non-zero values in all cells on the human cerebral organoid cells (Camp data [30]) as the candidate gene pool, and then chose the expression of overlap genes between the candidate genes and the PPI network/pathways to generate simulation data including noise data items at low-expression and high-expression levels respectively.

**Fig. 3:**
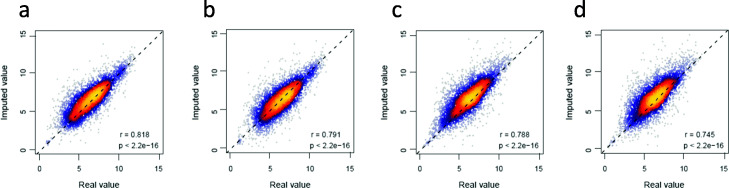
The scatter plots of imputation in the simulated data. **a**–**b** The PPI network-based imputation for the noise data at the low-expression and high-expression levels, respectively. **c**–**d** The pathway-based imputation for the noise data at the low-expression and high-expression levels, respectively

To simulate the low-expression data noise in scRNA-seq data, we randomly selected 5,000 expression values from the selected genes’ expression data, which had real values with high confidence tested by the Chebyshev inequality theorem as mentioned above, and replaced their expression values with zeros to introduce the dropouts and low-expression noise data. Conversely, to simulate the high-expression data noise in scRNA-seq data, we randomly selected 5,000 expression values from the gene expression data that had high confidence values, and replaced them with two times of the maximum value in the raw data to introduce the high-expression noise data. Based on the simulation data, we used the NetImpute method to recover the real values of replaced data items in each type of simulated datasets. Figure 3(a-b) show the scatter plots of the comparison between the real values of replaced items and their estimated values by incorporating gene interactions in the PPI network. As shown in Fig. 3(a-b), we can see that there are high correlations between the PPI network-based estimation data and the real values in raw data at both low-expression (*r*=0.818,*p*<2.2*e*−16) and high-expression (*r*=0.791,*p*<2.2*e*−16) levels. Meanwhile, Fig. 3(c-d) show the scatter plots of the comparison between the real values of replaced items and the estimated values of them at the low-expression and high-expression levels by incorporating the gene pathway information. As shown in Fig. 3(c-d), we also see that there are high correlations between the pathway-based estimation data and the real values at both the low-expression (*r*=0.788,*p*<2.2*e*−16) and high-expression (*r*=0.745,*p*<2.2*e*−16) levels. In conclusion, these correlation analyses demonstrate that the proposed NetImpute method can estimate the noise data items accurately by incorporating the biological network information. We also noticed that the estimation accuracy of noise data on the simulated data based on PPI network genes is slightly higher than the estimation based on pathway genes. The reason of this may be that the PPI network is overall larger than the pathway network and incorporates more interaction information among genes.

**Fig. 4:**
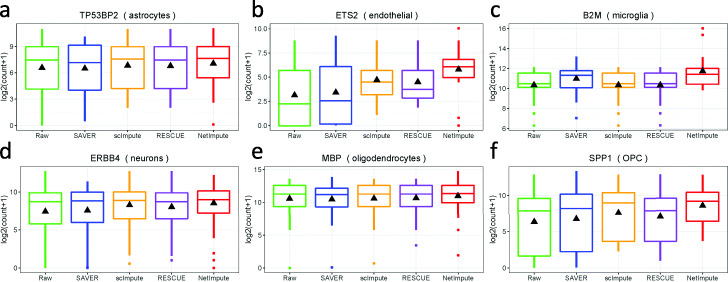
Examples of imputed expression distributions of genes that have cell-type specific functions in human brain neuronal cell types. The NetImpute imputed gene expression have better distribution patterns in cell types (have relatively centralized distribution in the same cell types)

In addition, to further illustrate the superiority of NetImpute on imputation of data noise in scRNA-seq data, we compared the data distribution of genes by using different imputation methods of scRNA-seq data, including the popular methods-SAVER [17], scImpute [4] and RESCUE [22]. Specifically, we deem that a better imputation method can estimate gene expression that has more significant discrimination to sense specific cell types from scRNA-seq data. Based on the human brain cells data (Darmanis data [31]), we selected the genes which have been reported having cell-type specific function characters in neuronal cells’ diversity to investigate their expression distribution in different cell types by using various imputation methods. For example, the gene TP53BP2 was reported as one of the astrocytes cell-type specific genes in terms of biological functions [45], while the MBP gene was reported as one of the oligodendrocytes cell-type marker genes [31, 45], et al. Figure 4 shows the imputed expression distributions in diverse cell-types’ samples of six example genes, which have cell-type specific functions in brain neuronal cells. As shown in Fig. 4, comparing with other reference methods, the NetImpute imputed data have more similar expression distribution on the cells of corresponding cell types. This indeed demonstrates that the proposed NetImpute method can accurately recover the real values of noise data items, thus to reveal more meaningful biomarkers in sensing of different cell types.

### Imputation based on PPI network improves the identification of cell types

In order to test whether the imputation of NetImpute based on the PPI network can help to improve the accuracy of identifying cell types from scRNA-seq data, we compared the accuracy performance of cell-types identification using different imputation methods on the three scRNA-seq data. Specifically, we compared the performance of NetImpute with four reference methods, including non-imputed data and imputed data by SAVER [17], scImpute [4] and RESCUE [22]. Since NetImpute needs to input the PPI network information, for the fairness of comparison, we used the overlap genes between the raw scRNA-seq data and the PPI network in all experiments (data details are shown in Table 1). In NetImpute, it automatically determined the number of principal components (PCs) in PCA by analysing each data before the initially fuzzy clustering (Camp data: 4 PCs; Darmanis data: 6 PCs; Li data: 7 PCs). As recommended above, in both scImpute and NetImpute methods, we set the cluster number of cell types as the true number in the pre-clustering procedure (Camp data: 5; Darmanis data: 8; Li data: 9). The parameters in other methods used their default values. Based on the imputed data, we used the hierarchical clustering algorithm to identify cell types from each data by cutting the dendrogram based on different distance heights. To evaluate the accuracy performance of cell-types identification, we calculated the adjusted Rand index (ARI [46]) and the normalized mutual information (NMI [47]) measurements between the predicted cell types and the annotated cell types.

Let *U*={*u*_1_,*u*_2_,...,*u*_*p*_} to denote the true cell-type labels in *p* clusters, *V*={*v*_1_,*v*_2_,...,*v*_*k*_} to denote the predicted cell types in *k* clusters. *n* is the total number of cells. The overlap between *U* and *V* can be summarized in a contingency table. The ARI can be calculated as [12, 46]

8 $$ ARI (U,V)=\frac{\sum_{i=1}^{p} \sum_{j=1}^{k} \binom{n_{ij}}{2}-[\sum_{i=1}^{p} \binom{a_{i}}{2}\sum_{j=1}^{k} \binom{b_{j}}{2}]/\binom{n}{2}}{\frac{1}{2}[\sum_{i=1}^{p}\binom{a_{i}}{2}+\sum_{j=1}^{k}\binom{b_{j}}{2}]-[\sum_{i=1}^{p}\binom{a_{i}}{2}\sum_{j=1}^{k} \binom{b_{j}}{2}]/\binom{n}{2}}  $$

where *n*_*ij*_ denotes the number of overlap cells between *u*_*i*_ and *v*_*j*_ (*n*_*ij*_=|*u*_*i*_∩*v*_*j*_|), *a*_*i*_ is the sum of the *i*-th row in the contingency table, *b*_*j*_ is the sum of the *j*-th column in the contingency table.

The NMI is defined as follows [48],

9 $$ NMI (U,V)= \frac{2I (U,V)}{H (U)+H (V)}  $$

where *I*(*U*,*V*) is the mutual information between *U* and *V*. It is defined as

10 $$ I (U,V)= \sum_{i=1}^{p}\sum_{j=1}^{k}\frac{ | u_{i}\cap v_{j} |}{n}log\frac{n | u_{i}\cap v_{j} |}{ | u_{i} |\times | v_{j} |}  $$

*H*(*U*) and *H*(*V*) are the entropy of *U* and *V* respectively. It is defined as

11 $$ H (U)=-\sum_{i=1}^{p}\frac{u_{i}}{n}log\frac{u_{i}}{n}, H (V)=-\sum_{j=1}^{k}\frac{v_{j}}{n}log\frac{v_{j}}{n}  $$

Since the number of predicted cell types depends on the distance parameter of the dendrogram cutting in hierarchical clustering, to obtain more accurate cell-types prediction for each method, we used different distance parameters to predict cell types and selected the best one which obtained the highest ARI performance as the final prediction parameter for each method.

**Fig. 5:**
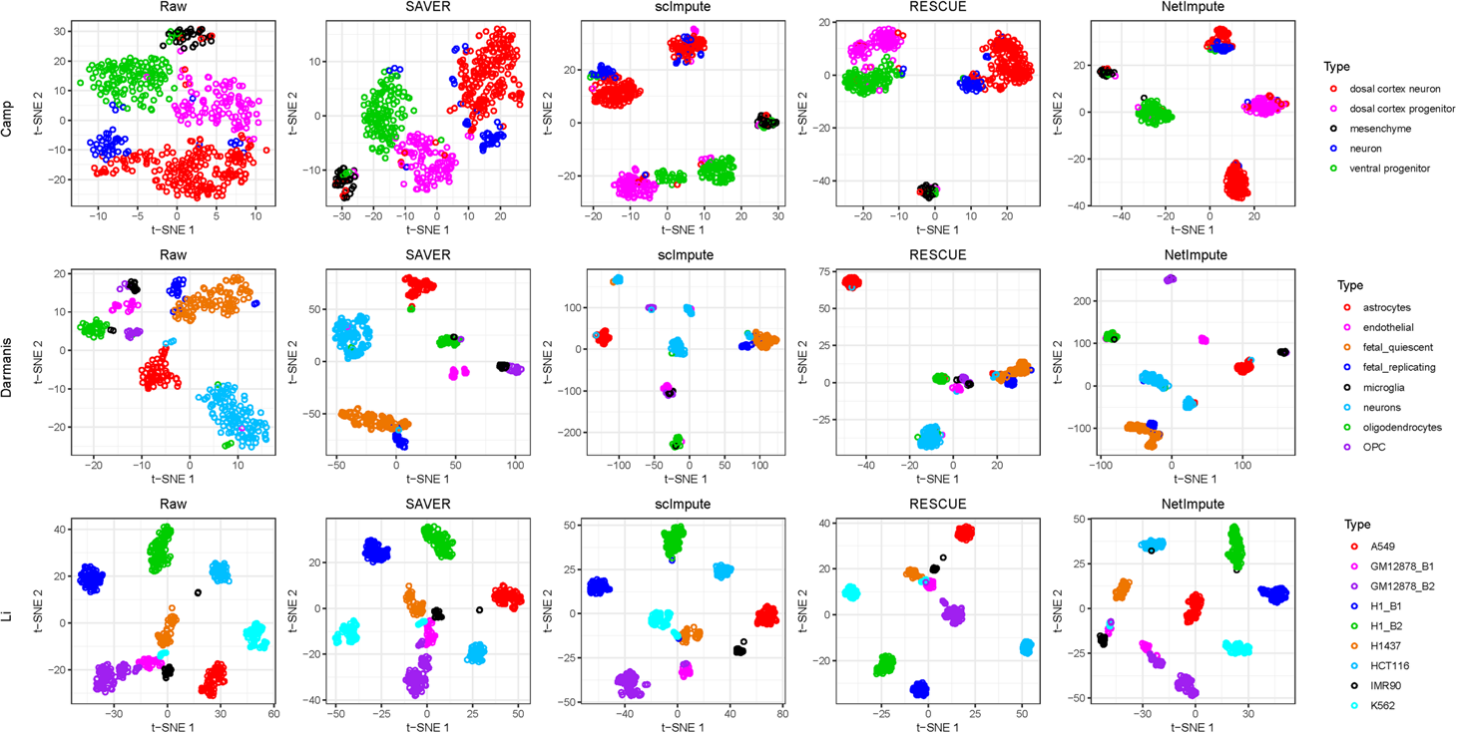
Visualization of the imputed data based on the PPI network genes. Three scRNA-seq data were used in experiments and different cell types were denoted with different colors

**Fig. 6:**
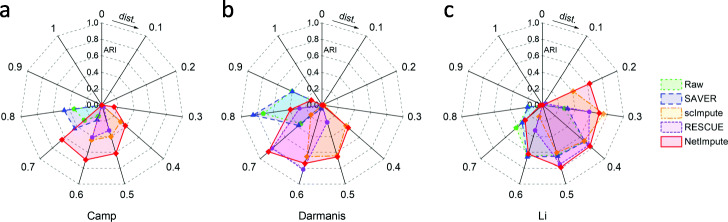
The ARI performance of cell-types prediction based on the PPI network-induced data by using different distance parameters of clustering in three datasets

**Fig. 7:**
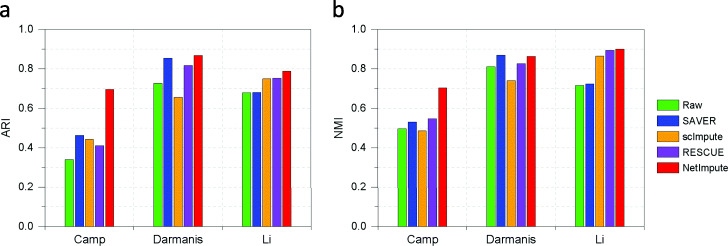
The cell-types prediction performance based on the PPI network-induced data using different methods in three datasets (the best parameter for each method was used in corresponding data). **a** The ARI performance of cell-types prediction. **b** The NMI performance of cell-types prediction

Figure 5 shows the visualization of cell-type clusters through t-SNE [49] to do dimension reduction on the processed data by using different methods. As shown in Fig. 5, we can see that the data imputed by the PPI-based NetImpute method tend to give more dense data distribution in intra-clusters of cell types and improve the quality of cell-types separation on most data comparing with other reference methods. For example, on Camp data, NetImpute can separate the dosal cortex progenitor and ventral progenitor cell types almost perfectly, while other methods cannot. To compare the cell-types prediction performance of each method in experiments, we used various distance height parameters in hierarchical clustering based on the imputed data and selected the best cell-types prediction labels as the final results in comparison. Figure 6 shows the ARI performance of cell-types prediction of each method using different parameters on each data. We can see that the imputed data using the PPI-based NetImpute method tend to give more accurate cell-types prediction on most parameters. This illustrates that the NetImpute method can help to improve the identification of cell types from scRNA-seq data. Figure 7(a-b) show the ARI and NMI performance of cell-types prediction using different imputation methods on three data. As shown in Fig. 7, the imputed data using the PPI-based NetImpute obtain better performance on cell-types identification on most experimental data. This indeed demonstrates that integrating the gene association information in PPI network can recover more accurate expression values of noise data in scRNA-seq data and thus to improve the identification of cell types from scRNA-seq data.

**Fig. 8:**
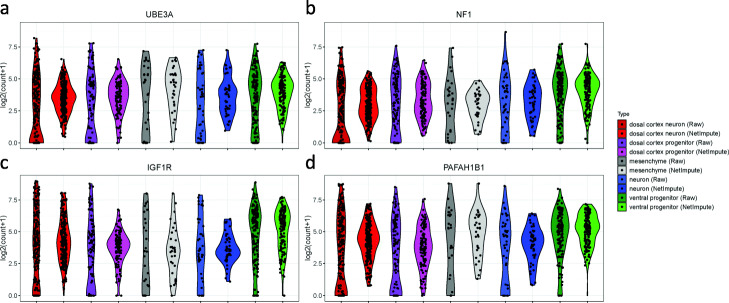
The violin plots of four cell-differentiation related genes to brain neuronal cells (Camp data). The expression levels of these genes are parts of different cell types in differentiation

In order to further illustrate the superiority of PPI-based NetImpute in cell-types identification, we investigated the expression distribution of the annotated cell-differentiation genes on human cerebral organoid. Figure 8 shows the expression violin plots of four example genes that related to cell-differentiation on Camp data [30]. The Camp data includes five cell-differentiation related cell types on cells. To be consistent with the functional differential of genes, we investigated the expression variation patterns of those genes in cell-differentiation cell types. Specifically, the UBE3A [50], NF1 [51, 52], IGF1R [53] and PAFAH1B1 [54] genes are reported to be related to the development of brain neuronal cells. As shown in Fig. 8, the PPI-based NetImpute imputed data reveal better expressed variability patterns of related genes in the differentiation of cell types. In conclusion, the PPI-based NetImpute can impute the scRNA-seq data accurately and thus enhance the identification of cell types in practice.

### Imputation based on pathways improves the identification of cell types

**Fig. 9:**
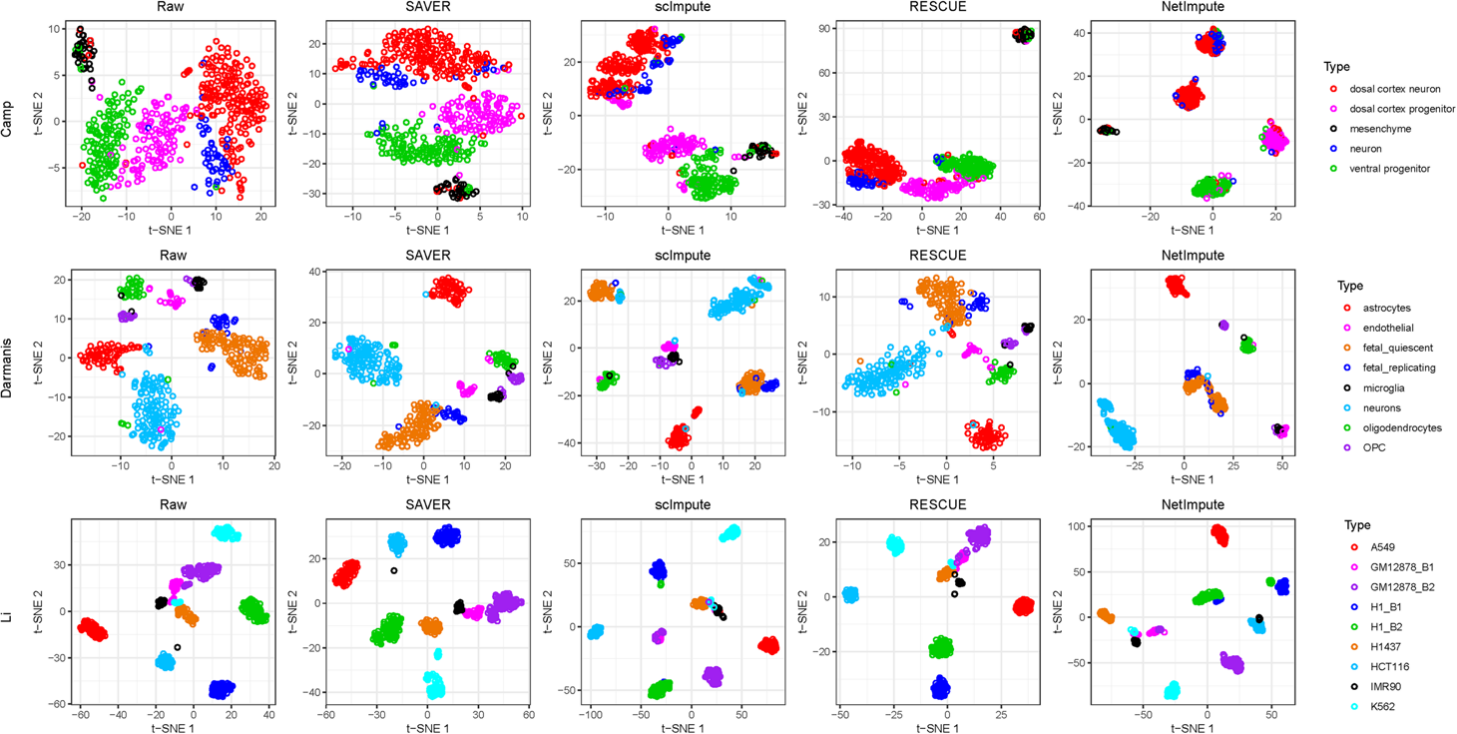
Visualization of the imputed data based on the pathway genes. Three scRNA-seq data were used in experiments and different cell types were denoted with different colors

**Fig. 10:**
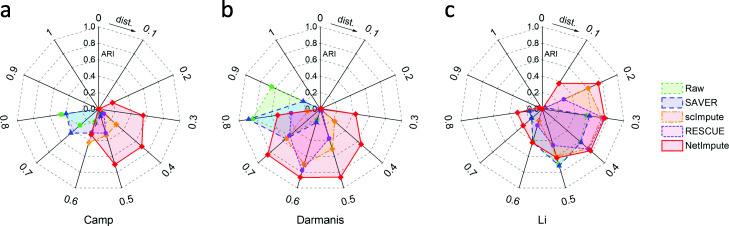
The ARI performance of cell-types prediction based on the pathway-induced data by using different distance parameters of clustering in three datasets

**Fig. 11:**
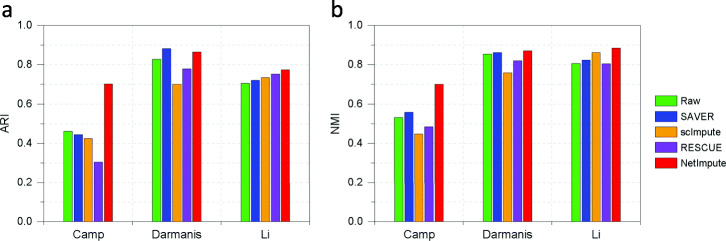
The cell-types prediction performance based on the pathway-induced data using different methods in three datasets (the best parameter for each method was used in corresponding data experiments). **a** The ARI performance of cell-types prediction. **b** The NMI performance of cell-types prediction

We also tested whether the imputation of NetImpute based on gene pathways can help to improve the identification of cell types from scRNA-seq data. Similar to the experiments on the PPI-based imputation, we used the overlap genes between the raw scRNA-seq data and gene pathways (Table 1) and also used the same analysis pipeline and parameter setting methods in experiments. Figure 9 shows the visualization of cell-type clusters using dimension reduction of t-SNE [49] on the pathway-based processed data by using different methods. We can see that the data imputed by the pathway-based NetImpute method tend to give more dense data distribution in intra-clusters and improve the quality of cell-types separation on most data compared with other reference methods. Specifically, it separates the endothelial, oligodendrocytes and OPC cell types more clearly than other methods on the Darmanis data. To further evaluate the performance of the identification of cell types based on the imputed data, we used different distance height cutting parameters in hierarchical clustering to identify the best cell-types prediction of different methods and compared their performance on different data. Figure 10 shows the ARI performance on cell-types prediction of each method based on different clustering parameters on each data. We can also see that the imputed data using the pathway-based NetImpute method tend to give more accurate cell-types prediction on most clustering parameters comparing with other methods. Figure 11(a-b) show the ARI and NMI performance on cell-types prediction using different imputation methods on the three data. As shown in Fig. 11, the imputed data using the pathway-based NetImpute method obtain better performance on cell-types identification on most experimental data. This indeed demonstrates that integrating the gene association information in pathways can estimate more accurate gene expression of noise items and thus to improve the identification of cell types from scRNA-seq data.

### Fusion of imputed data is more powerful in identifying cell types

**Fig. 12:**
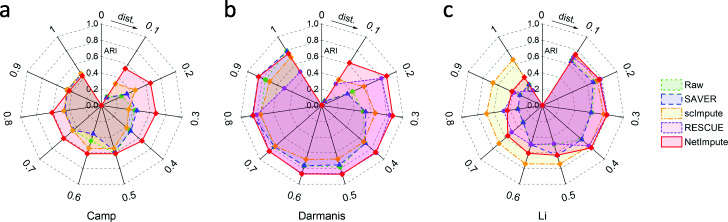
The ARI performance of cell-types prediction based on the fused imputation data (PPI-based and pathway-based) by using different distance parameters in three datasets

One of the main contributions in this work is that we fuse the imputed data based on the PPI network and gene pathways to identify cell types from scRNA-seq data. In order to demonstrate the advantage of data fusion in cell-types identification, we compared the performance of cell-types prediction on each individual imputed data and the fused imputed data respectively. In detail, since the reference imputation methods cannot integrate the PPI network and pathway information to impute scRNA-seq data, to be fair for the methods comparison in our experiments, we only used the expression data of genes in the PPI network/pathways which intersect with the gene set in raw scRNA-seq data. Based on the integrated distance information across cells, we used the hierarchical clustering algorithm to identify cell types by using different dendrogram cutting parameters. Figure 12 shows the ARI performance on cell-types prediction of each method using different distance height cutting parameters. We can see that the NetImpute method has better prediction performance than other methods under most parameters on Camp and Darmanis data; while on the Li data, both scImpute and NetImpute have better prediction performance than others, although scImpute looks better than NetImpute under most parameters. We set the clustering parameter on each type of imputed data as the one which obtained the best ARI performance. Tables 2 and 3 show the ARI and NMI performance on the cell-types prediction in terms of different imputation methods and data sources. As Tables 2 and 3 shown, the NetImpute method obtains better prediction performance than others not only on the individual data but also on the fusion data in most data conditions. This demonstrates the NetImpute imputation method can help to improve the identification of cell types. In addition, we also notice that the fused imputation data based on the PPI network and gene pathways can help further enhance the identification of cell types to most reference methods. This demonstrates that integrating multiple types of prior biological data can effectively improve the identification of cell types from scRNA-seq data.

**Table 2:** The ARI performance of cell-types prediction on different types of imputation data in three datasets. The PPI-based, pathway-based and fused imputation data were used in comparison

Dataset	Camp			Darmanis			Li		
	PPI	Pathway	Fusion	PPI	Pathway	Fusion	PPI	Pathway	Fusion
Raw	0.341	0.462	0.612	0.726	0.828	0.794	0.678	0.705	0.708
SAVER	0.464	0.445	0.578	0.854	**0.881**	0.858	0.680	0.720	0.680
scImpute	0.443	0.425	0.545	0.655	0.701	0.758	0.750	0.733	0.754
RESCUE	0.411	0.305	0.554	0.816	0.778	**0.879**	0.752	0.753	**0.799**
NetImpute	**0.695**	**0.702**	**0.673**	**0.868**	0.865	0.873	**0.788**	**0.773**	0.792

**Table 3:** The NMI performance of cell-types prediction on different types of imputation data in three datasets. The PPI-based, pathway-based and fused imputation data were used in comparison

Dataset	Camp			Darmanis			Li		
	PPI	Pathway	Fusion	PPI	Pathway	Fusion	PPI	Pathway	Fusion
Raw	0.496	0.532	0.642	0.811	0.853	0.771	0.715	0.806	0.850
SAVER	0.530	0.558	0.643	**0.869**	0.862	0.847	0.723	0.823	0.840
scImpute	0.486	0.447	0.565	0.739	0.759	0.791	0.865	0.862	0.863
RESCUE	0.548	0.485	0.588	0.827	0.819	0.850	0.893	0.805	**0.919**
NetImpute	**0.704**	**0.701**	**0.664**	0.863	**0.871**	**0.872**	**0.900**	**0.884**	0.909

## Discussion

The advance of scRNA-seq technologies provides a great opportunity to investigate the transcriptional variability characteristics at the single-cell resolution. However, the dropout events and high background noise are big challenges in scRNA-seq data analyses at present, although many imputation models have been proposed over the past years. Identification of cell types is one of the most important research purposes on scRNA-seq analyses to reveal the transcriptional variability among cells. The NetImpute framework integrates multiple types of biological networks to impute scRNA-seq data accurately and fuses different types of imputation data to identify cell types automatically. To handle the dropout events in cell-types clustering, although there are many statistic tools available at present, most of them only consider the data noise at low-expression level, such as SAVER [17], scImpute [4], RESCUE[22] and CIDR [19], etc. However, the large amounts of data noise in scRNA-seq data can also occur at high-expression level since the technical biases [10, 11]. There are few methods can handle the high-expression noise in scRNA-seq data currently. The NetImpute imputation model uses a statistic method to detect data noise at both low-expression and high-expression levels and imputes the noise data items by integrating gene association information in the PPI network and gene pathways. This method overcomes the limitation of current methods that they cannot handle the noise data items at high-expression level. In addition, integrating the prior gene association information in biological networks to impute the scRNA-seq data provides a new idea to handle high-noisy scRNA-seq data to obtain more accurate estimation data. Besides, the NetImpute integration model automatically fuses multiple imputation data to identify cell types. It provides a new framework to integrate multiple types of biological information to improve the identification of cell types on the downstream analyses of scRNA-seq data.

One of the most important features in the NetImpute imputation model is that users need to specify the number of fuzzy cell subpopulations *K* before running the imputation algorithm. Similar to scImpute [4], it can be selected by referring the clustering result of the raw data or the cluster number of user estimate based on the prior data knowledge. The selection of *K* determines the cell subpopulation units in fitting of imputation model. Although we use the fuzzy clustering method to detect the preliminary cell subpopulations in NetImpute to ease the effect of the parameter, we still recommend to set it close to the true number of cell types in raw data since the NetImpute performs imputation based on the cell samples in the same subpopulations. Another important feature is the parameters of *θ*_1_ and *θ*_2_ in Eq.2, which control the thresholds of variation to determine the noise data items at the low-expression and high-expression levels, respectively. Large values of these two parameters lead to low proportion imputation of data noise. In this study, considering the large rate of dropout events at low-expression level and relatively small rate of noise at high-expression level in raw scRNA-seq data, we set *θ*_1_=0 and *θ*_2_=0.5 in default. In general, users can set the parameters to control the rate of imputation in practice.

In the NetImpute integration model, we only fused the imputed data based on the PPI network and gene pathways to identify cell types at present. Using more complete and accurate association information between genes may help to improve performance on cell-types prediction. Although we used the relatively complete PPI network in this study, the noise in the PPI network may also affect the imputation model’s learning. More accurate PPI network with considering the noise associations and other types of biological networks, such as the gene regulatory network, etc., can also be used to extend NetImpute framework. In addition, we used the linear regression model to impute the noise items at present. The non-linear prediction model hopes to consider more complex expression associations among genes and may give more accurate imputations. Besides, in the data fusion model, we simply concatenated the distance matrixes from multiple types of data to identify cell types according to hierarchical clustering. The complicated similarity network fusion methods may hope to be used to further improve the accuracy of cell-types prediction in future.

## Conclusions

We propose an integrated framework, NetImpute, to identify cell types from scRNA-seq data by incorporating biological networks to impute the raw noise data items and fusing multiple types of imputation data. Comprehensive experiments on three real scRNA-seq data demonstrate that: (1) The proposed biological network-based imputation model can estimate more accurate scRNA-seq data, and the data imputed by NetImpute is helpful to improve the identification of cell types and reveal the expression patterns of genes in cell types. (2) The proposed integration model based on multiple types of imputation data has better performance on the identification of cell types from scRNA-seq data. In conclusion, the NetImpute model provides a new framework to identify cell types from scRNA-seq data by integrating multiple types of biological networks. We hope the NetImpute would be a useful approach to analyse scRNA-seq data in future.

## Data Availability

All data materials used in this study were collected from public datasets. The source code is available at https://github.com/yiangcs001/NetImpute.

## Abbreviations

ARI: Adjusted rand index
FCM: Fuzzy c-means
NMI: Normalized mutual information
NNLS: Non-negative least squares
PCA: Principal component analysis
PCC: Pearson correlation coefficient
PCs: Principal components
PPI: Protein-protein interaction
scRNA-seq: Single-cell RNA sequencing
t-SNE: T-distributed stochastic neighbor embedding
